# The stereochemical course of 2-methylisoborneol biosynthesis

**DOI:** 10.3762/bjoc.18.82

**Published:** 2022-07-08

**Authors:** Binbin Gu, Anwei Hou, Jeroen S Dickschat

**Affiliations:** 1 Kekulé-Institute of Organic Chemistry and Biochemistry, University of Bonn, Gerhard-Domagk-Straße 1, 53121 Bonn, Germany

**Keywords:** biosynthesis, enantioselective synthesis, enzyme mechanisms, gas chromatography, terpenoids

## Abstract

Both enantiomers of 2-methyllinalyl diphosphate (2-Me-LPP) were synthesized enantioselectively using Sharpless epoxidation as a key step and purification of enantiomerically enriched intermediates through HPLC separation on a chiral stationary phase. Their enzymatic conversion with 2-methylisoborneol synthase (2MIBS) demonstrates that (*R*)-2-Me-LPP is the on-pathway intermediate, while a minor formation of 2-methylisoborneol from (*S*)-2-Me-LPP may be explained by isomerization to 2-Me-GPP and then to (*R*)-2-Me-LPP.

## Introduction

After its first discovery from *Streptomyces* [1–2], it has been recognized that many soil bacteria including various genera from the actinobacteria [3–7] and myxobacteria [8] produce the volatile musty odour compound 2-methylisoborneol (**1**). The compound is also found in marine *Streptomyces* strains [9] and aquatic cyanobacteria that can cause drinking water contaminations in water supply systems [10–11]. In addition, the liverwort *Lophocolea heterophylla* [12] and various strains of *Penicillium* [13] have been reported as a source of compound **1**. As a consequence of cheese fermentation with *Penicillium*, compound **1** can add to the flavor of Camembert and Brie [14], but in other foodstuff such as fish and coffee contaminations with **1** are perceived as unpleasant flavor constituents [15–18]. Despite its occurrence in fungi, **1** also has moderate antifungal activity as observed for its inhibition of mycelial growth and sporulation in *Fusarium moniliforme* [19]. Recent research on its chemical ecology demonstrated that arthropodes are attracted by compound **1** which helps in the dispersion of *Streptomyces* spores [20].

**Scheme 1:**
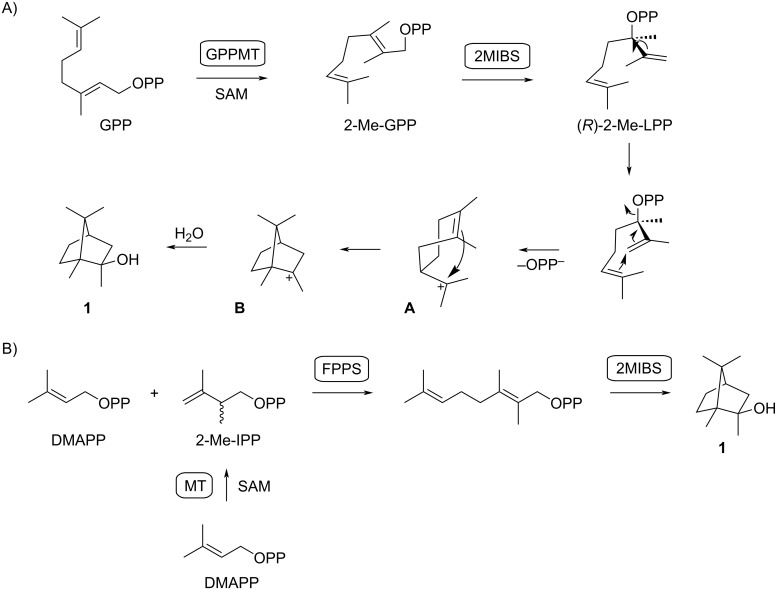
Biosynthesis of 2-MIB (**1**). A) Naturally observed pathway through methylation of GPP to 2-Me-GPP by GPPMT and cyclization to **1** by 2MIBS. B) Reconstituted pathway through methylation of DMAPP to 2-Me-IPP with a hypothetical MT, followed by coupling of DMAPP and 2-Me-IPP to 2-Me-GPP by FPPS and cyclization to **1** by 2MIBS.

The absolute configuration of (–)-**1** has been established through a synthesis from (+)-camphor [21]. The biosynthesis of compound **1** was initially suggested to proceed through degradation of a sesquiterpene [2], but first feeding experiments with ^14^C-labeled acetate and methionine pointed to a methylated monoterpene [22]. Further investigations by feeding of ^13^C-labeled methionine and deuterated mevalonolactone isotopomers to *Nannocystis exedens* resulted in a biosynthetic model that includes the methylation of geranyl diphosphate (GPP) to 2-methyl-GPP (2-Me-GPP), followed by cyclization to compound **1** (Scheme 1A) [8]. This process involves the isomerization of 2-Me-GPP by allylic transposition of diphosphate to 2-methyllinalyl diphosphate (2-Me-LPP), followed by a conformational change through rotation around the C2–C3 bond and cyclization to the 2-methyl-α-terpinyl cation (**A**). A second cyclization to **B** and attack of water results in 2-methylisoborneol (**1**) [8]. The stereochemical details of this cyclization cascade were first suggested by Cane, with processing through (*R*)-2-Me-LPP [23]. The GPP methyltransferase (GPPMT) and the 2-methylisoborneol synthase (2MIBS) and their coding genes were discovered and functionally characterized, giving further evidence for the biosynthetic pathway to compound **1** [23–25]. As we have recently demonstrated, the biosynthesis of **1** can also be reconstituted in vitro through coupling of dimethylallyl diphosphate (DMAPP) with 2-methyl-IPP (2-Me-IPP; IPP = isopentenyl diphosphate) to 2-Me-GPP using farnesyl diphosphate synthase (FPPS), followed by cyclization through 2MIBS to **1** [26]. A recently described methyltransferase from *Micromonospora humi* can convert DMAPP into (*R*)-2-Me-IPP with a methyltransferase [27], naturally providing the C_6_ building block for this hypothetical alternative pathway towards **1** (Scheme 1B).

**Figure 1:**
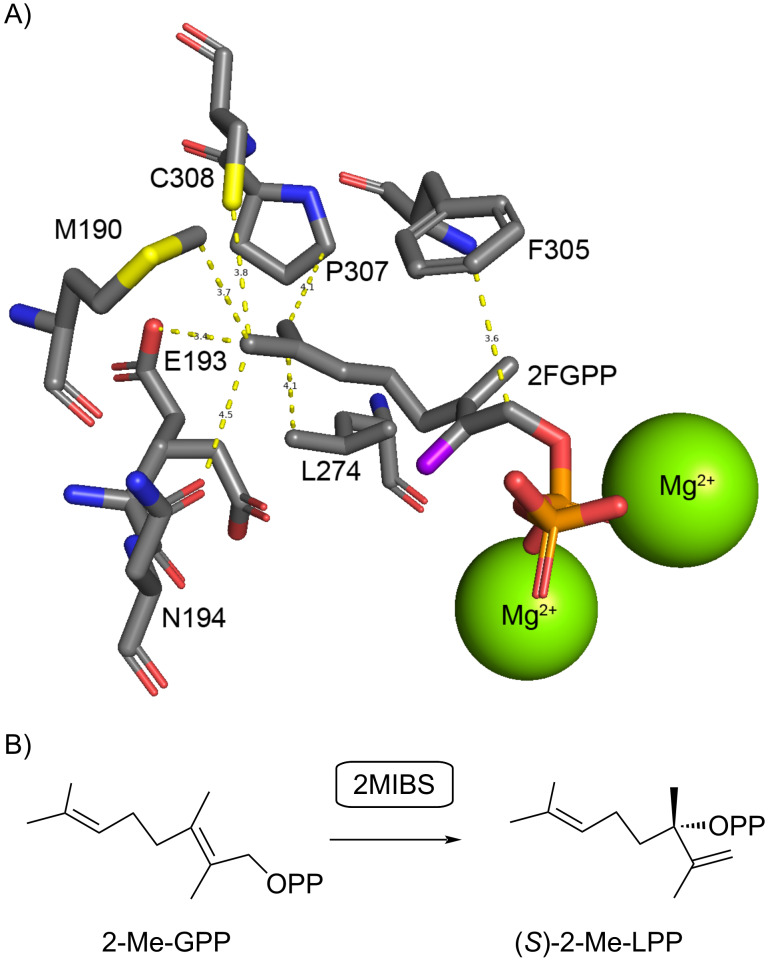
A) Active site of 2MIBS with the bound substrate surrogate 2FGPP (generated with Pymol from the crystal structure, PDB code: 3V1X). B) Isomerization of 2-Me-GPP to (*S*)-2-Me-LPP, the hypothetical enantiomer expected for 2-Me-GPP in the same conformation as observed for 2FGPP.

Today the genomes of many bacteria from the genus *Streptomyces* have been made available, showing that the genes for the biosynthesis of **1** are present in about half of the species [28], which is reflected by the frequent detection of **1** among the volatiles emitted by a large number of streptomycetes and closely related bacteria [3–7]. A series of side products of the 2MIBS has been identified by GC/MS analysis and synthesis of reference compounds [29], several of which also occur in *Escherichia coli* or yeast strains that were engineered for the biosynthesis of methylated monoterpenes derived from 2-Me-GPP [30–31]. About one decade ago, the crystal structures of GPPMT and 2MIBS have been solved [32–33]. Notably, the structure of 2MIBS has been obtained in complex with the non-reactive substrate analog 2-fluoro-GPP (2FGPP), showing the substrate surrogate in a stretched conformation in the active site of 2MIBS (Figure 1A). The observed conformation of 2FGPP, if this is also relevant for the native substrate 2-Me-GPP, seems to imply that the isomerization through suprafacial allylic transposition of diphosphate should result in the intermediate (*S*)-2-Me-LPP (Figure 1B), which would be the opposite enantiomer as suggested by Cane [23]. This prompted us to investigate whether (*R*)- or (*S*)-2-Me-LPP is the true pathway intermediate towards compound **1**. For this purpose, both enantiomers of 2-Me-LPP were synthesized and enzymatically converted by 2MIBS. Here we report on the enantioselective synthesis of (*R*)- and (*S*)-2-Me-LPP and the results from the incubation experiments with 2MIBS.

## Results and Discussion

### Enantioselective synthesis of 2-methyllinalyl diphosphate

**Scheme 2:**
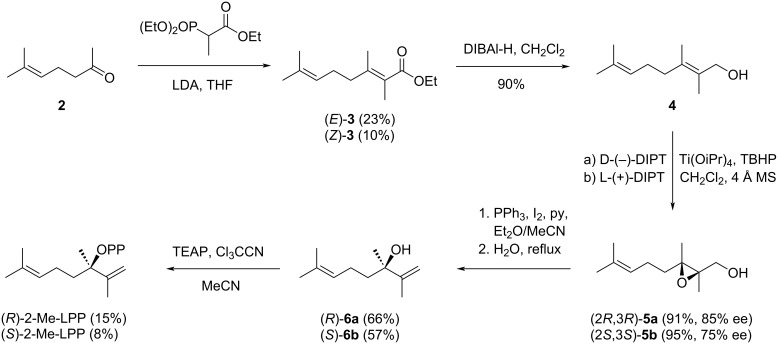
Synthesis of (*R*)- and (*S*)-2-Me-LPP.

The synthesis of (*R*)- and (*S*)-2-Me-LPP started with the Horner–Wadsworth–Emmons reaction [34–35] of sulcatone (**2**) with triethyl 2-phosphonopropionate to obtain ethyl 2-methylgeranate (**3**) as a mixture of the *E* and *Z* stereoisomers (5:2) that were separated by column chromatography (Scheme 2). Reduction of (*E*)-**3** with DIBAl-H gave 2-methylgeraniol (**4**) that was converted under Sharpless conditions [36] into the epoxides (2*R*,3*R*)-**5a** using ᴅ-(−)-diisopropyl tartrate (DIPT) and (2*S*,3*S*)-**5b** with ʟ-(−)-DIPT. The enantiomeric purity of both compounds was determined by small scale conversions with (*S*)-α-methoxy-α-trifluoromethylphenylacetyl chloride (Mosher’s acid chloride) [37] and ^1^H NMR analysis of the products (Figure S1 in Supporting Information File 1), showing enantiomeric purities of 85% ee for **5a** and 75% ee for **5b**. Further conversion by treatment with PPh_3_, iodine, pyridine, and water [38] gave access to (*R*)- and (*S*)-2-methyllinalool (**6a** and **6b**). The materials were subsequently converted into the diphosphates using triethylammonium phosphate and trichloroacetonitrile [39] (Scheme 2).

### Conversion of enantiomerically enriched 2-Me-LPP with 2-MIBS

**Figure 2:**
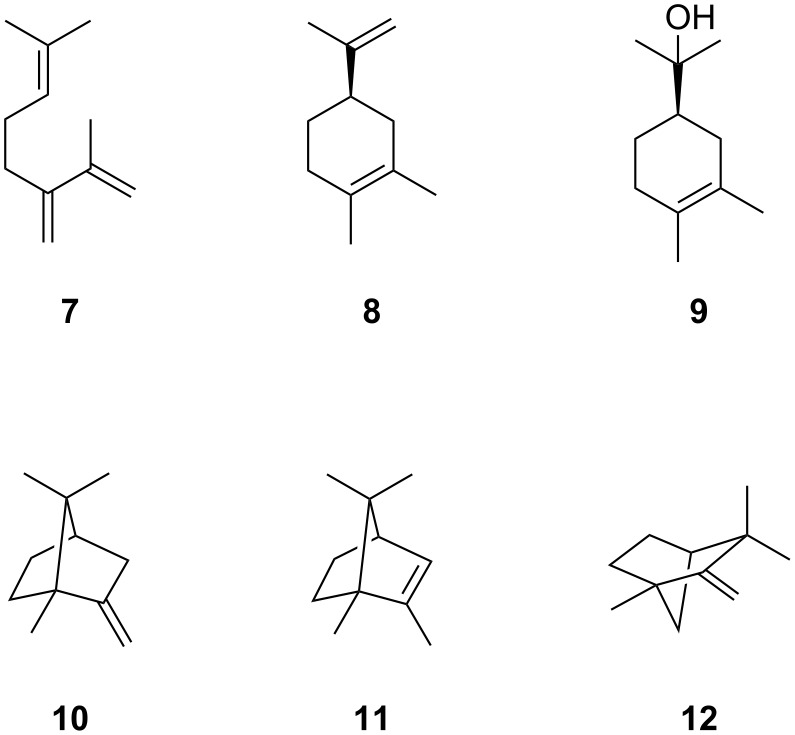
Structures of 2MIBS side products and spontaneous degradation products of 2-Me-LPP. The enantiomers shown are the expected on-pathway intermediates towards **1**.

The enantiomerically enriched substrates (*R*)- and (*S*)-2-Me-LPP were incubated with purified 2MIBS (Figure S2 in Supporting Information File 1), followed by extraction of the enzyme reactions with hexane and GC/MS analysis of the obtained products (Figure S3, Table S1 in Supporting Information File 1). All compounds were identified from their EI mass spectra and retention indices in comparison to synthetic standards [29]. The substrate (*R*)-2-Me-LPP gave high yields of compound **1** (62% of total enzyme products in GC), besides 2-methylenebornane (**10**, 21%) and small amounts of 2-methylmyrcene (**7**, 4%), 2-methyllimonene (**8**, 1%), 2-methyl-α-terpineol (**9**, 9%), 2-methyl-2-bornene (**11**, 1%), and 2-methylenefenchane (**12**, 2%). In contrast, (*S*)-2-Me-LPP yielded compound **9** as the main product (51%) and minor amounts of **1** (31%), besides **7** (8%), **8**, (1%), **10** (6%), **11** (0.3%), and **12** (0.6%) (structures are shown in Figure 2). Reproducibility of these results was demonstrated in triplicates. While these data showed that enantiomerically enriched (*R*)-2-Me-LPP is more efficiently converted into **1** than the enriched *S* enantiomer, the enantiomeric purity of the substrates was not sufficiently high to decide, if only one enantiomer of LPP serves as the precursor to **1**.

### Purification of the enantiomers of 2-Me-LPP

In order to obtain the enantiomers of 2-Me-LPP with high purity, the synthetically obtained enantiomerically enriched compounds **6a** and **6b** were purified by HPLC using a chiral stationary phase. Ultimately, enantiomeric purities of >99% ee were reached for both **6a** and **6b** (Figure S4, Supporting Information File 1). The compounds were subsequently converted into the diphosphates. To exclude partial racemization during this conversion, small samples of each enantiomer of 2-Me-LPP were dephosphorylated with calf intestinal phosphatase (CIP). The thus obtained compounds **6a** and **6b** were analyzed by gas chromatography on a chiral stationary phase, revealing that the materials were unchanged and still of very high enantiomeric purity (>99% ee, Figure S5 in Supporting Information File 1).

### Conversion of enantiomerically pure 2-Me-LPP with 2-MIBS

**Figure 3:**
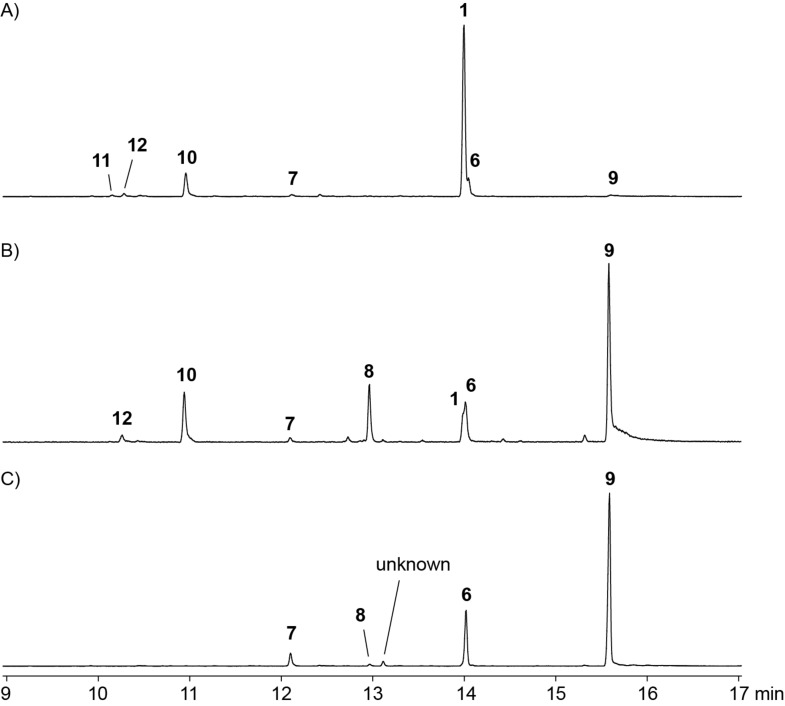
Total ion chromatograms of extracts from an incubation of A) enantiomerically pure (*R*)-2-Me-LPP with 2-MIBS, B) enantiomerically pure (*S*)-2-Me-LPP with 2-MIBS, and C) enantiomerically pure (*R*)-2-Me-LPP without enzyme in incubation buffer. The result for (*S*)-2-Me-LPP without enzyme in incubation buffer was the same as in C) and is not shown.

Both pure enantiomers of 2-Me-LPP were incubated with 2-MIBS. GC/MS analysis of the products (Figure 3A and 3B, and Table S1 in Supporting Information File 1) showed with the substrate (*R*)-2-Me-LPP an efficient conversion into 2-methylisoborneol (**1**, 75%). Minor compounds included 2-methylenebornane (**10**, 13%), 2-methyllinalool (**6**, 8%), 2-methylenefenchane (**12**, 2%), 2-methyl-2-bornene (**11**, 1%), 2-methyl-α-terpineol (**9**, 1%), and 2-methylmyrcene (**7**, 1%). In contrast, (*S*)-2-Me-LPP yielded mainly **9** (55%), but only small amounts of **1** coeluting with **6** (sum: 15%). Further minor products included **10** (13%), **8** (13%), **12** (2%), and **7** (1%). Control experiments by incubation of (*R*)- and (*S*)-2-Me-LPP in buffer containing no enzyme revealed a major non-enzymatic formation of **9** (70%), besides smaller amounts of **6** (22%) and **7** (5%) next to traces of **8** (1%) and another unknown compound (2%, Figure 3C). Reproducibility of these data was again shown in triplicates.

**Scheme 3:**
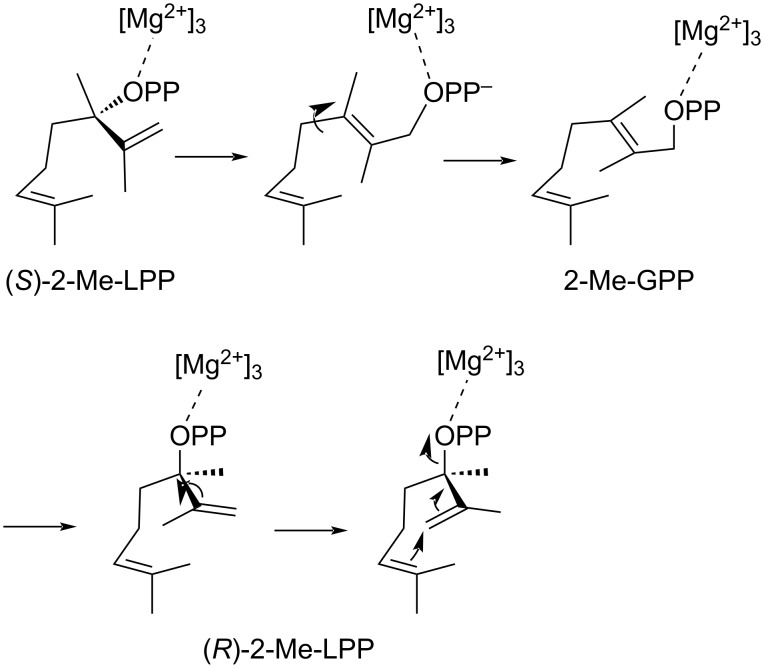
Hypothetical mechanism for the isomerization of (*S*)-2-Me-LPP through 2-Me-GPP to (*R*)-2-Me-LPP.

These results demonstrate that (*R*)-2-Me-LPP is the on-pathway intermediate to compound **1**. However, the small amounts of **1** formed from (*S*)-2-Me-LPP (>99% ee) are too large to be explained from the minor enantiomer (*R*)-2-Me-LPP in this sample (<1%). A possible explanation is that (*S*)-2-Me-LPP can bind to the active site of 2MIBS in a non-productive conformation. Its enzyme assisted isomerization to 2-Me-GPP followed by a conformational change may allow for another isomerization to (*R*)-2-Me-LPP and thus lead to the observed minor formation of **1** (Scheme 3). In contrast to the product distribution from (*R*)-2-Me-LPP with **1** as the main and **10** as a side product of 2MIBS, the hydrocarbon **10** is formed from (*S*)-2-Me-LPP in slightly larger amounts than **1**, which could be explained by an incorrect placing or incomplete binding of the active site water involved in the formation of **1**, if (*S*)-2-Me-LPP occupies the active site of 2MIBS.

The formation of **6** and **9** in the incubations of (*R*)- and (*S*)-2-Me-LPP with 2MIBS seems to be non-enzymatic in all cases, because the enantiomeric composition of these products is nearly the same for enzymatic and non-enzymatic reactions, as shown by GC on a chiral stationary phase (Supporting Information File 1, Figures S6 and S7), minor participation of the enzyme in the formation of these products cannot be excluded). In contrast, the formation of **8** from (*S*)-2-Me-LPP must involve the participation of 2MIBS, because its production is clearly enhanced in comparison to the non-enzymatic sample. Investigation of the enantiomeric composition through GC on a chiral stationary phase reveals a 2:3 ratio of enantiomers in favor of (*R*)-**8** (Figure S8, Supporting Information File 1), suggesting that a partial isomerization of (*S*)-2-Me-LPP to (*R*)-2-Me-LPP according to Scheme 3 is also relevant for the formation of **8**. The pseudoracemic mixture of the synthetic compounds **6a** and **6b**, as well as enantiomerically enriched synthetic reference compounds for **8** and **9** (Scheme S1 in Supporting Information File 1) were used for comparison in these analyses.

## Conclusion

Both enantiomers of 2-Me-LPP can be selectively prepared using a Sharpless epoxidation strategy in high enantiomeric purity of >75% ee. HPLC purification of the synthetic precursor 2-methyllinalool using a chiral stationary phase can make the pure enantiomers available, and their conversion into the enantiomers of 2-Me-LPP proceeds without noticeable racemization, leading to materials of >99% ee. Incubation of both pure enantiomers revealed that (*R*)-2-Me-LPP is the on-pathway intermediate towards **1**, while its formation from (*S*)-2-Me-LPP may be explained through isomerization to 2-Me-GPP and then to (*R*)-2-Me-LPP. Conclusively, these findings confirm Cane’s mechanistic proposal [23], while the observed conformation of 2FGPP in the crystal structure of 2MIBS may not represent the required conformation for 2-Me-GPP for the production of **1**.

## Supporting Information

File 1 Experimental.
