# Supplementary material for: The stereochemical course of 2-methylisoborneol biosynthesis
Source: Beilstein J Org Chem. 2022 Jul 8;18:818–24. doi: 10.3762/bjoc.18.82 (PMC9273983; doi:10.3762/bjoc.18.82)
Supplement: File 1 — Experimental. [file Beilstein_J_Org_Chem-18-818-s001.pdf]

BEILSTEIN JOURNAL OF ORGANIC CHEMISTRY

Supporting Information

for

The stereochemical course of 2-methylisoborneol biosynthesis

Binbin Gu, Anwei Hou and Jeroen S. Dickschat

Beilstein J. Org. Chem. **2022,** *18,* 818–824. doi:10.3762/bjoc.18.82

Experimental

License and Terms: This is a supporting information file under the terms of the Creative Commons Attribution License (https://creativecommons.org/licenses/by/4.0). Please note that the reuse, redistribution and reproduction in particular requires that the author(s) and source are credited and that individual graphics may be subject to special legal provisions.
The license is subject to the *Beilstein Journal of Organic Chemistry* terms and conditions: (https://www.beilstein-journals.org/bjoc/terms)

General
Chemicals were purchased from Sigma Aldrich Chemie GmbH (Steinheim, Germany), Carbolution Chemicals GmbH (St. Ingbert, Germany), or Carl Roth (Karlsruhe, Germany) and used without purification. Solvents for column chromatography were purchased in p.a. grade and purified by distillation. Thin-layer chromatography was performed with 0.2 mm precoated plastic sheets Polygram Sil G/UV254 purchased from Machery-Nagel (Düren, Germany). Column chromatography was performed using silica gel 60 (0.040–0.060 nm) purchased from Merck (Darmstadt, Germany).

GC/MS
GC/MS analyses were carried out on a 7890B/5977A series gas chromatography/mass selective detector (Agilent, Santa Clara, CA, USA). The GC was equipped with an HP5-MS fused silica capillary column (30 m, 0.25 mm ID, 0.50 µm film; Agilent) and operated using the settings: 1) inlet pressure: 77.1 kPa, He at 23.3 mL min^{-1}, 2) injection volume: 1 µL, 3) temperature program: 5 min at 50 °C then increasing 10 °C min^{-1} to 320 °C, 4) splitless or split ratio 50:1, 60 s valve time, and 5) carrier gas: He at 1 mL min^{-1}. The MS was operated with settings: 1) source: 230 °C, 2) transfer line: 250 °C, 3) quadrupole: 150 °C, and 4) electron energy: 70 eV. Retention indices (*I*) were determined from a homologous series of *n*-alkanes (C_7–C_{40}).

NMR spectroscopy
NMR spectra were recorded on a Bruker Avance I 500 MHz spectrometer and a Bruker Avance III HD 700 MHz Cryo spectrometer. Chemical shifts were referenced to the residual proton signal of C_6D_6 (δ = 7.16 ppm) for ^{1}H NMR and the ^{13}C signal of C_6D_6 (δ = 128.06 ppm) for ^{13}C NMR [1].

HRMS
High resolution mass spectra were recorded with LTQ Orbitrap XL (Thermo Fisher Scientific, Waltham. Massachusetts, USA).

Optical rotations
Optical rotations were recorded on a Modular Compact Polarimeter MCP 100 (Anton Paar, Graz, Austria). The temperature setting was 20 °C; the wavelength of the light used was 589 nm (sodium D line); the path-length was 10 cm; the compound concentrations *c* are given in g 100 mL^{-1}.

Synthesis of (*R*) and (*S*)-2-Me-LPP

The synthesis of (*R*)- and (*S*)-2-Me-LPP was performed as outlined in Scheme 2 of main text.

Synthesis of ethyl (*E*)- and (*Z*)-2,3,7-trimethylocta-2,6-dienoate (3)

Diisopropylamine (12.0 g, 118.9 mmol, 1.5 equiv) was dissolved in THF (400 mL) and cooled to 0 °C. Then a solution of *n*-BuLi (1.6 M in hexane, 74.5 mL, 118.9 mmol, 1.5 equiv) was added dropwise and stirring was continued for 1 h. The reaction mixture was cooled to −78 °C, triethyl 2-phosphonopropionate (28.5 g, 118.9 mmol, 1.5 equiv) was added, and the reaction mixture was stirred for 2 h, before 6-methylhept-5-en-2-one (**2**, 10.0 g, 79.3 mmol, 1.0 equiv) was added dropwise at the same temperature. The reaction mixture was stirred at 65 °C overnight. The reaction was quenched by the addition of water (400 mL) and extracted with Et_2O (3 × 400 mL). The combined organic layers were dried with $MgSO_4$, the solvent was removed under reduced pressure, and the crude product was purified using silica gel column chromatography [*n*-pentane/Et_2O 20:1] to obtain the title compounds (*E*)-**3** (3.88 g, 18.5 mmol, 23%) and (*Z*)-**3** (1.69 g, 7.9 mmol, 10%) as colourless oils [2].

(*E*)-3: ^{1}H-NMR (500 MHz, C_6D_6): δ [ppm] = 5.14-5.08 (m, 1H, CH), 4.05 (q, *J* = 7.1 Hz, 2H, CH_2), 2.10 (q, *J* = 1.5 Hz, 3H, CH_3), 2.03 (brd, *J* = 3.4 Hz, 4H, 2 x CH_2), 1.92 (q, *J* = 1.5 Hz, 3H, CH_3), 1.62 (d, *J* = 1.2 Hz, 3H, CH_3), 1.50 (d, *J* = 1.3 Hz, 3H, CH_3), 1.01 (t, *J* = 7.1 Hz, 3H, CH_3). ^{13}C-NMR (126 MHz, C_6D_6): δ [ppm] = 169.1 (C_q), 146.4 (C_q), 132.0 (C_q), 124.1 (CH), 123.4 (C_q), 59.8 (CH_2), 36.5 (CH_2), 26.3 (CH_2), 25.8 (CH_3), 21.3 (CH_3), 17.6 (CH_3), 15.6 (CH_3), 14.4 (CH_3). TLC [*n*-pentane/Et_2O (20:1)]: R_f = 0.35. GC (HP-5MS): *I* = 1454. MS (EI, 70 eV): *m*/*z* (%) = 210 (3), 165 (23), 142 (24), 137 (24), 136 (11), 96 (43), 69 (100), 67 (24), 53 (13), 41 (50).

(*Z*)-3: ^{1}H-NMR (500 MHz, C_6D_6): δ [ppm] = 5.32-5.26 (m, 1H, CH), 4.04 (q, J = 7.1 Hz, 2H, CH_2), 2.58-2.52 (m, 2H, CH_2), 2.31-2.23 (m, 2H, CH_2), 1.83 (q, *J* = 0.9 Hz, 3H, CH_3), 1.67 (d, *J* = 1.3 Hz, 3H, CH_3), 1.62 (brs, 3H, CH_3), 1.54 (q, *J* = 0.9 Hz, 3H, CH_3), 1.01 (t, *J* = 7.1 Hz, 3H, CH_3). ^{13}C-NMR (126 MHz, C_6D_6): δ [ppm] = 168.9 (C_q), 146.2 (C_q), 131.7 (C_q), 124.8 (CH), 123.6 (C_q), 59.9 (CH_2), 36.9 (CH_2), 27.7 (CH_2), 25.9 (CH_3), 20.4 (CH_3), 17.7 (CH_3), 16.0 (CH_3), 14.4 (CH_3). TLC [*n*-pentane/Et_2O (20:1)]: R_f = 0.45. GC (HP-5MS): *I* = 1421. MS (EI, 70 eV): *m*/*z* (%) = 210 (3), 165 (13), 142 (24), 137 (31), 96 (51), 69 (100), 67 (33), 53 (19), 41 (68).

Synthesis of (*E*)-2,3,7-trimethylocta-2,6-dien-1-ol (4)

The ester (*E*)-**3** (3.88 g, 18.5 mmol, 1.0 equiv) was dissolved in THF (56 mL) and cooled to 0 °C. DIBAL-H (1 M in hexane, 44.4 mL, 44.4 mmol, 2.4 equiv) was added slowly and the reaction mixture was stirred at 0 °C for 1 h. The reaction was quenched by adding saturated sodium potassium tartrate solution (100 mL) and the mixture was extracted with Et_2O (3 × 300 mL). The organic phases were dried with $MgSO_4$, the solvent was removed, and the crude product was subjected to column chromatography on silica gel [*n*-pentane/Et_2O 2:1] to obtain the alcohol **4** (2.80 g, 16.7 mmol, 90%) as colourless oil [2].

4: ^{1}H-NMR (500 MHz, C_6D_6): δ [ppm] = 5.22-5.18 (m, 1H, CH), 3.95 (s, 2H, CH_2), 2.12-2.02 (m, 4H, 2 x CH_2), 1.73 (d, *J* = 1.5 Hz, 3H, CH_3), 1.66 (d, *J* = 1.3 Hz, 3H, CH_3), 1.59 (d, *J* = 1.5 Hz, 3H, CH_3), 1.55 (s, 3H, CH_3). ^{13}C-NMR (126 MHz, C_6D_6): δ [ppm] = 131.7 (C_q), 131.5 (C_q), 129.0 (C_q), 124.8 (CH), 63.8 (CH_2), 35.3 (CH_2), 26.9 (CH_2), 25.9 (CH_3), 17.9 (CH_3), 17.7 (CH_3), 16.2 (CH_3). TLC [*n*-pentane/Et_2O (2:1)]: R_f = 0.32. GC (HP-5MS): *I* = 1328. MS (EI, 70 eV): *m*/*z* (%) = 168 (5), 153 (9), 150 (7), 137 (11), 135 (7), 107 (13), 98 (19), 94 (11), 85 (13), 82 (28), 81 (21), 79 (12), 69 (100), 67 (28), 55 (28), 53 (21), 43 (90), 41 (86), 39 (27).

Synthesis of (2*R*,3*R*)- and (2*S*,3*S*)-(3-(4-methylpent-3-en-1-yl)-2,3-dimethyloxiran-2-yl)methanol (5a and 5b)

In a literature-known procedure [3], diisopropyl D-(−)-tartrate (180 mg, 0.77 mmol, 0.13 equiv) and Ti(OiPr)$_4$ (185 mg, 0.65 mmol, 0.11 equiv) were dissolved in CH_2Cl_2 (20 mL) and the mixture was cooled to −20 °C. A solution of alcohol **4** (1.0 g, 5.94 mmol, 1.0 equiv) in CH_2Cl_2 (10 mL) was added dropwise, before *tert*-butyl hydroperoxide (5.5 M in decane, 2.3 mL, 2.1 equiv) was added. The reaction mixture was stirred at −20 °C for 2 h and hydrolysed by the addition of 10% tartaric acid solution (10 mL). The reaction mixture was stirred for 2 h without cooling. The organic phase was

washed with H_2O (50 mL) and the aqueous phase was extracted with CH_2Cl_2 (3 × 30 mL). The combined organic layers were dried with $MgSO_4$ and the solvent was removed under reduced pressure. The crude product was dissolved in Et_2O (30 mL) and cooled to 0 °C, followed by the addition of 1 M NaOH solution (15 mL). The reaction mixture was stirred for 40 min, the phases were separated and the aqueous phase was extracted with Et_2O (3 × 15 mL). The combined organic phases were washed with saturated NH_4Cl solution and brine followed by drying with $MgSO_4$. The solvent was removed under reduced pressure. Column chromatography on silica gel [petroleum ether/EtOAc 1:1] resulted in the epoxy alcohol (2*R*,3*R*)-**5a** (1.0 g, 5.43 mmol, 91%, 85% ee) that was obtained as a colourless oil.
The same procedure was used to convert the alcohol **4** (1.0 g, 5.94 mmol, 1.0 equiv) into (2*S*,3*S*)-**5b** (1.05 g, 5.67 mmol, 95%, 75% ee) using diisopropyl L-(+)-tartrate. Mosher ester analyses [4] for the determination of the enantiomeric excesses were performed by dissolving 5 µL of each epoxy alcohol in $CDCl_3$ (400 µL). Then, pyridine (5 µL) and (*S*)-MTPA-Cl (5 µL) were added. The reaction mixture was stirred at room temperature for 2 h and the corresponding Mosher ester products were purified by column chromatography on silica gel [petroleum ether/ethyl acetate 10:1] and analysed by ^{1}H NMR (Figure S1).
(2*R*,3*R*)-5a: ^{1}H-NMR (500 MHz, C_6D_6): δ [ppm] = 5.14-5.09 (m, 1H, CH), 3.51 (dd, *J* = 11.4, 4.5 Hz, 1H, 0.5 x CH_2), 3.46 (dd, *J* = 11.5, 5.8 Hz, 1H, 0.5 x CH_2), 2.13-1.96 (m, 2H, CH_2), 1.77 (t, *J* = 5.7 Hz, 1H, OH), 1.67-1.61 (m, 1H, 0.5 x CH_2), 1.63 (d, *J* = 1.2 Hz, 3H, CH_3), 1.52-1.45 (m, 1H, 0.5 x CH_2), 1.51 (d, *J* = 1.4 Hz, 3H, CH_3), 1.32 (s, 3H, CH_3), 1.20 (s, 3H, CH_3). ^{13}C-NMR (126 MHz, C_6D_6): δ [ppm] = 131.7 (C_q), 124.3 (CH), 65.6 (CH_2), 64.6 (C_q), 64.5 (C_q), 35.9 (CH_2), 25.8 (CH_3), 24.6 (CH_2), 18.0 (CH_3), 17.6 (CH_3), 16.4 (CH_3). TLC [petroleum ether/EtOAc (1:1)]: R_f = 0.43. GC (HP-5MS): *I* = 1379. MS (EI, 70 eV): *m*/*z* (%) = 151 (2), 135 (2), 123 (5), 111 (19), 110 (19), 109 (21), 102 (13), 95 (50), 93 (18), 85 (17), 84 (16), 83 (16), 82 (13), 81 (12), 75 (58), 71 (21), 69 (100), 67 (43), 57 (44), 55 (27), 53 (18), 43 (94), 41 (88), 39 (34). HRMS (TOF): *m*/*z* = 183.1390 (calc. for $[C_{11}H_{19}O_2]^-$ 183.1391). $[\alpha]_D^{20}$ = +5.6° (*c* 1.3, CH_2Cl_2).
(2*S*,3*S*)-5b: HRMS (TOF): *m*/*z* = 183.1388 (calc. for $[C_{11}H_{19}O_2]^-$ 183.1391). $[\alpha]_D^{20}$ = –4.0° (*c* 1.1, CH_2Cl_2). Spectroscopic data as for the (2*R*,3*R*) enantiomer.

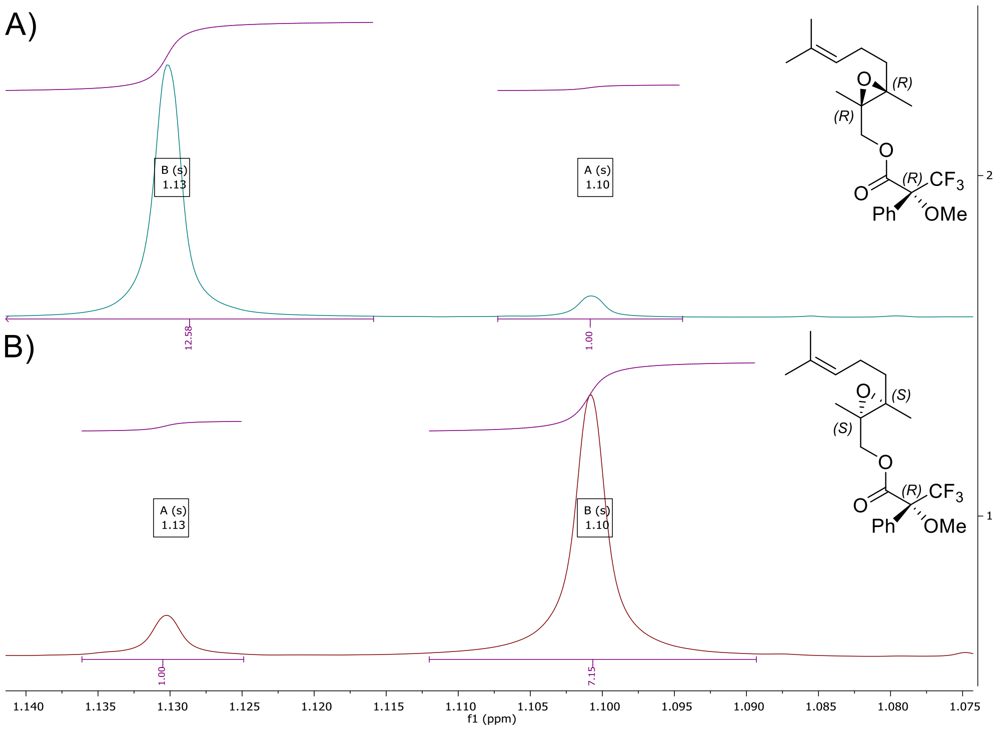

Figure S1: Mosher ester analysis of **5a** and **5b**. Partial ^{1}H NMR spectra of the reaction product of (*S*)-MTPA-Cl with A) (2*R*,3*R*)-**5a** and B) (2*S*,3*S*)-**5b** showing the signal for Me-3. Integration gave an approximated enantiomeric excess of 85% for (2*R*,3*R*)-**5a** and 75% for (2*S*,3*S*)-**5b**.

Synthesis of (*R*)- and (*S*)-2,3,7-trimethylocta-1,6-dien-3-ol (6a and 6b)

Following a known procedure [3], (2*R*,3*R*)-**5a** (500 mg, 2.71 mmol, 1.0 equiv) was dissolved in Et_2O/CH_3CN 5:3 (21.5 mL). The solution was cooled to 0 °C and PPh_3 (2.13 g, 8.13 mmol, 3.0 equiv), pyridine (857 mg, 10.84 mmol, 4.0 equiv), and I_2 (1.03 g, 4.07 mmol, 1.5 equiv) were added sequentially. Stirring was continued for 2 h at 0 °C and then H_2O (48.6 µL, 2.71 mmol, 1.0 equiv) was added. The reaction mixture was heated to reflux for 12 h with stirring. The reaction was quenched by the addition of saturated $Na_2S_2O_3$ solution (5.4 mL) and saturated aqueous $NaHCO_3$ solution (5.4 mL). The aqueous phase was extracted with Et_2O (3 × 20 mL), and the combined organic layers were washed with 0.5 M HCl (10 mL) and saturated aqueous $NaHCO_3$ solution (10 mL), dried with $MgSO_4$, and concentrated under reduced pressure. Column chromatography on silica gel [petroleum ether/EtOAc 10:1] resulted in the alcohol (*R*)-**6a** (300 mg, 1.78 mmol, 66%) that was obtained as a colourless oil.

The same procedure was used to convert (2*S*,3*S*)-**5b** (500 mg, 2.71 mmol, 1.0 equiv) into (*S*)-**6b** (260 mg, 1.55 mmol, 57%).

(*R*)-6a: ^{1}H-NMR (500 MHz, C_6D_6): δ[ppm] = 5.22-5.16 (m, 1H, CH), 5.11-5.07 (m, 1H, 0.5 x CH_2), 4.83-4.81 (m, 1H, 0.5 x CH_2), 2.16-2.04 (m, 1H, 0.5 x CH_2), 2.04-1.92 (m, 1H, 0.5 x CH_2), 1.65 (q, *J* = 1.3 Hz, 3H, CH_3), 1.62 (dd, *J* = 1.5, 0.7 Hz, 3H, CH_3), 1.55 (d, *J* = 1.3 Hz, 3H, CH_3), 1.58-1.51 (m, 2H, CH_2), 1.15 (s, 3H, CH_3), 1.07 (s, 1H, OH). ^{13}C-NMR (126 MHz, C_6D_6): δ[ppm] = 150.8 (C_q), 131.3 (C_q), 125.3 (CH), 109.8 (CH_2), 75.2 (C_q), 40.7 (CH_2), 28.1 (CH_3), 25.9 (CH_3), 23.2 (CH_2), 19.5 (CH_3), 17.7 (CH_3). TLC [petroleum ether/EtOAc (10:1)]: R_f = 0.36. GC (HP-5MS): *I* = 1194. MS (EI, 70 eV): *m*/*z* (%) = 150 (15), 135 (24), 125 (12), 109 (15), 107 (55), 94 (31), 86 (46), 85 (77), 83 (19), 79 (18), 71 (27), 69 (58), 67 (29), 57 (70), 55 (46), 53 (17), 43 (76), 41 (100), 39 (41). HRMS (TOF): *m*/*z* = 167.1439 (calc. for $[C_{11}H_{19}O]^-$ 167.1441). $[\alpha]_D^{20}$ = –17.3° (*c* 1.2, CH_2Cl_2).

(*S*)-6b: HRMS (TOF): *m*/*z* = 167.1438 (calc. for $[C_{11}H_{19}O]^-$ 167.1441). $[\alpha]_D^{20}$ = +17.0° (*c* 1.0, CH_2Cl_2). Spectroscopic data as for the (*R*) enantiomer.

Synthesis of (*R*) and (*S*)-2-Me-LPP

In a direct phosphorylation procedure [5,6], bis-triethylammonium phosphate (TEAP) solution (2.0 mL, prepared by adding 3.64 mL of solution A (2.5 mL phosphoric acid, 9.4 mL MeCN) to 6 mL of solution B (11 mL triethylamine, 10 mL MeCN) was added to a solution of (*R*)-**6a** (100 mg, 0.59 mmol, 1.0 equiv) in CCl_3CN (2 mL) at room temperature. After 2 more additions of TEAP solution (2 × 2 mL) in 5 min intervals, the reaction mixture was purified by silica gel chromatography [iPrOH/25% NH_3/H_2O 6:2.5:0.5] to yield the diphosphate (*R*)-2-Me-LPP (34.5 mg, 0.09 mmol, 15%) as a white solid after lyphilisation.

Using the same conditions, (*S*)-**6b** (100 mg, 0.59 mmol, 1.0 equiv) was converted into (*S*)-2-Me-LPP (18 mg, 0.05 mmol, 8%).

(*R*)-2-Me-LPP: ^{1}H-NMR (500 MHz, D_2O): δ[ppm] = 5.28-5.20 (m, 1H, CH), 5.06 (d, *J* = 1.7 Hz, 1H, 0.5 x CH_2), 4.98-4.93 (m, 1H, 0.5 x CH_2), 2.05-1.90 (m, 1H, 0.5 x CH_2), 1.90-1.83 (m, 1H, 0.5 x CH_2), 1.80 (s, 3H, CH_3), 1.79-1.67 (m, 1H, 0.5 x CH_2), 1.70 (s, 3H, CH_3), 1.65 (s, 3H, CH_3), 1.66-1.60 (m, 1H, 0.5 x CH_2), 1.63 (s, 3H, CH_3). ^{13}C-NMR (126 MHz, D_2O): δ[ppm] = 148.4 (d, $^3J_{C,P}$ = 4.8 Hz, C_q), 133.6 (C_q), 124.5 (CH_2), 111.3 (CH), 84.8 (d, $^2J_{C,P}$ = 7.6 Hz, C_q), 39.2 (d, $^3J_{C,P}$ = 5.8 Hz, CH_2), 24.9 (CH_3), 23.6 (d, $^3J_{C,P}$ = 1.4 Hz, CH_3), 22.6 (CH_2), 18.5 (CH_3), 17.0 (CH_3). ^{31}P-NMR (202 MHz, D_2O): δ[ppm] = –9.97 (d, $^2J_{P,P}$ = 20.2 Hz, 1P), –15.38 (d, $^2J_{P,P}$ = 21.4 Hz, 1P). HRMS (TOF): *m*/*z* = 327.0762 (calc. for $[C_{11}H_{21}O_7P_2]^-$ 327.0768).

(*S*)-2-Me-LPP: HRMS (TOF): *m*/*z* = 327.0763 (calc. for $[C_{11}H_{21}O_7P_2]^-$ 327.0768). Spectroscopic data as for the (*R*) enantiomer.

Incubation of enantiomerically enriched (*R*)-2-Me-LPP (or (*S*)-2-Me-LPP) in incubation buffer with 2MIBS

Culture conditions, protein expression, and protein purification were performed as described previously [7]. The soluble enzyme fraction was checked for purity by SDS-PAGE (Figure S2). The incubations were performed with 0.3 mg enantiomerically enriched (*R*)-2-Me-LPP (85% ee) or (*S*)-2-Me-LPP (75% ee) dissolved in substrate buffer (50 µL; 25 mM NH_4HCO_3) and diluted with incubation buffer (850 µL; 50 mM Tris/HCl, 10 mM $MgCl_2$, 10% glycerol, pH 8.2). 2MIBS protein solution (100 µL) obtained from 100 mL expression culture was added to the above solutions, followed by incubation with shaking at 30 °C overnight. The crude product was extracted with hexane (500 µL), the extracts were dried with $MgSO_4$, and directly analysed by GC/MS (Figure S3).

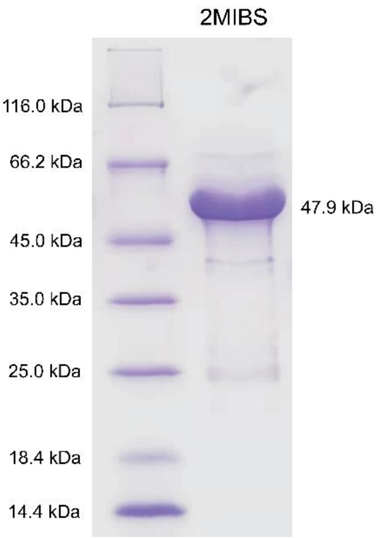

Figure S2: SDS-PAGE analysis of recombinant 2MIBS.

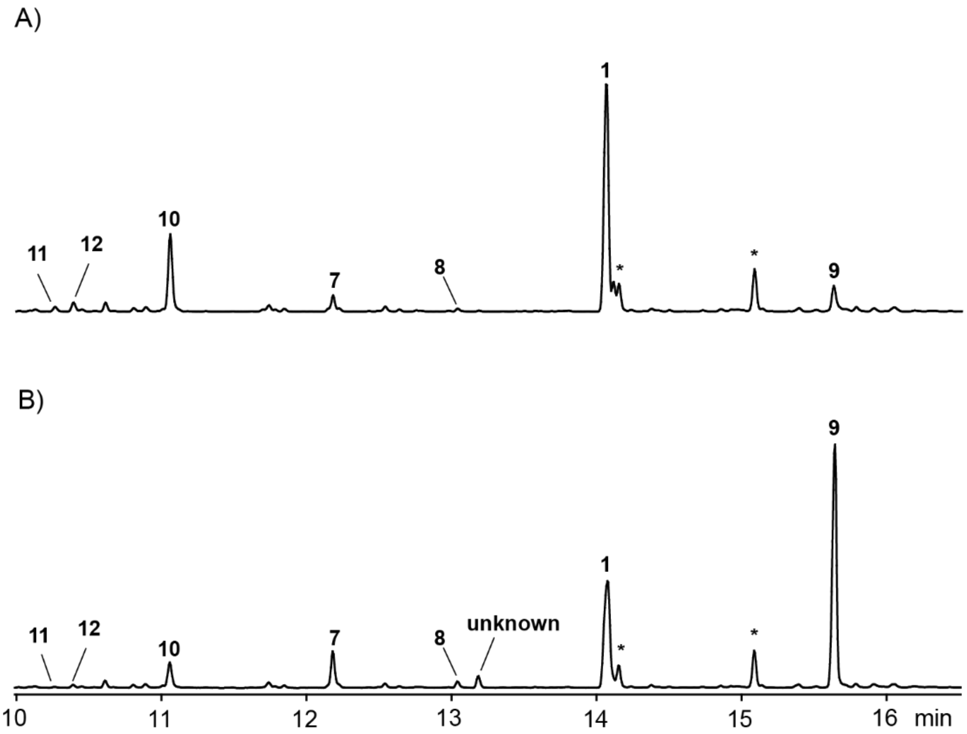

Figure S3: Total ion chromatograms of extracts from an incubation of A) enantiomerically enriched (*R*)-2-Me-LPP with 2MIBS, B) enantiomerically enriched (*S*)-2-Me-LPP with 2MIBS. Asterisks indicate contaminants.

Table S1. Identified enzyme products.

**Compound**[a]	***I***[b]	***I* (Lit.)**[c]	**Ident.**[d]
2-methyl-2-bornene (**11**)	986	977 [8]	ms, ri, std
2-methylenefenchane (**12**)	992	980 [8]	ms, ri, std
2-methylenebornane (**10**)	1025	1009 [8]	ms, ri, std
2-methylmyrcene (**7**)	1084	1080 [8]	ms, ri, std
2-methyllimonene (**8**)	1133	1125 [8]	ms, ri, std
2-methylisoborneol (**1**)	1194	1177 [8]	ms, ri, std
2-methyl-α-terpineol (**9**)	1297	1284 [8]	ms, ri, std

[a]Compound numbers refer to compound numbers in the main text. Unidentified compounds, artifacts, contaminants, and enzyme reaction buffer constituents are not mentioned. [b]Retention index on a HP5-MS fused silica capillary column. [c]Retention index on the same column from tabulated data in the literature. [d]Identification based on ms: mass spectrum (mass spectral match factor >850), ri: retention index on same column (maximum deviation of 17 points), std: comparison to a synthetic standard.

Purification of the enantiomers of 6 by HPLC using a chiral stationary phase

In order to obtain the pure enantiomers of 2-Me-LPP, the enantiomerically enriched synthetic compounds **6a** and **6b** were purified by HPLC on a chiral stationary phase.

For analytical separations a PLATINblue-series UHPLC system (Knauer, Berlin, Germany) was used, equipped with a photo diode array detector PDA-1 (190–1000 nm). Separation of **6a** and **6b** was performed using a DAICEL Chiralpak IG-U column (1.6 μm; 3.0 × 100 mm) and an isocratic solvent mixture of heptane/2-propanol 98:2 with a flow of 0.85 mL min^{-1} (328 bar). The UV–vis absorption was monitored at 210 nm. Observed elution times were 1.89 min (**6b**) and 2.57 min (**6a**) as shown in Figure S4.

Preparative scale HPLC purifications were performed on the same system with a PHENOMENEX i-Amylose 3 column (5 μm; 250 × 21 mm) connected with a pre-column (15 × 21.2 mm). For elution an isocratic mixture of heptane/2-propanol 98:2 at 18 mL min^{-1} (47 bar) was used.

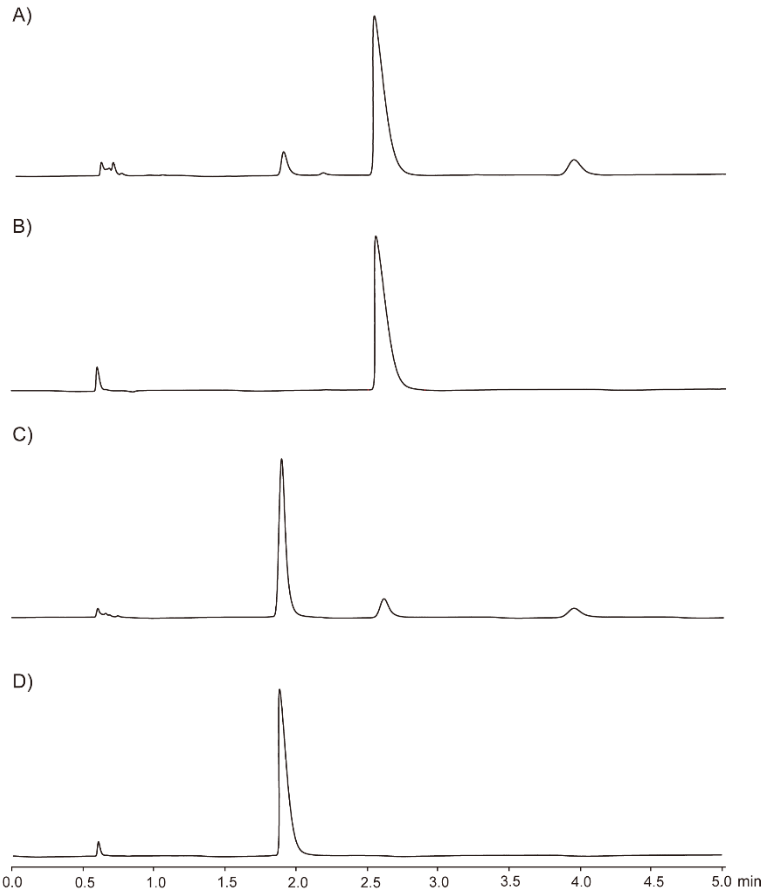

Figure S4: HPLC chromatograms of A) enantiomerically enriched **6a** before HPLC purification, B) enantiomerically pure **6a** after HPLC purification on a chiral stationary phase, C) enantiomerically enriched **6b** before HPLC purification, and D) enantiomerically pure **6b** after HPLC purification on a chiral stationary phase. The obtained purified compounds **6a** and **6b** were enantiomerically pure (>99% ee).

Conversion of enantiomerically pure 6a and 6b into (*R*)- and (*S*)-2-Me-LPP

The purified enantiomers of **6a** and **6b** were converted into (*R*)- and (*S*)-2-Me-LPP using the same method as described above for enantiomerically enriched **6a** and **6b**.

Incubation of enantiomerically pure (*R*)-2-Me-LPP (or (*S*)-2-Me-LPP) with calf intestinal phosphatase

To exclude a possible loss of enantiomeric purity during this transformation, small samples of the obtained compounds (*R*)- and (*S*)-2-Me-LPP were treated with calf intestinal phosphatase (CIP), followed by extraction with hexane, drying with $MgSO_4$, and analysis by GC on a chiral stationary phase. Specifically, the incubations were performed with 0.3 mg enantiomerically pure (*R*)-2-Me-LPP or (*S*)-2-Me-LPP dissolved in substrate buffer (50 µL; 25 mM NH_4HCO_3) and diluted with incubation buffer (940 µL; 50 mM Tris/HCl, 10 mM $MgCl_2$, 10% glycerol, pH 8.2). Then, calf intestinal phosphate (CIP, New England Biolabs, 10 units) and CutSmart Buffer (10 µL) were added and the above mixtures were incubated for 1 h at 37 °C. The mixture was extracted with hexane (500 µL), the extracts were dried, and subjected to Chiral GC analysis (Figure S5).

GC analysis using a chiral stationary phase

An Agilent GC 7820A GC equipped with an FID detector and an Agilent Cyclosil-B capillary column (30 m, 0.25 mm ID, 0.25 µm film) was used for GC analysis. For analysis of all the samples in this research work, the GC was programmed under the same conditions as follows: starting from 80 °C and holding this temperature for 5 min, increasing with 10 °C/min to 220 °C while holding this temperature for 5 min. Inlet temperature: 250 °C, injection volume: 1 µL, carrier gas: H_2 at 2.3 mL/min.

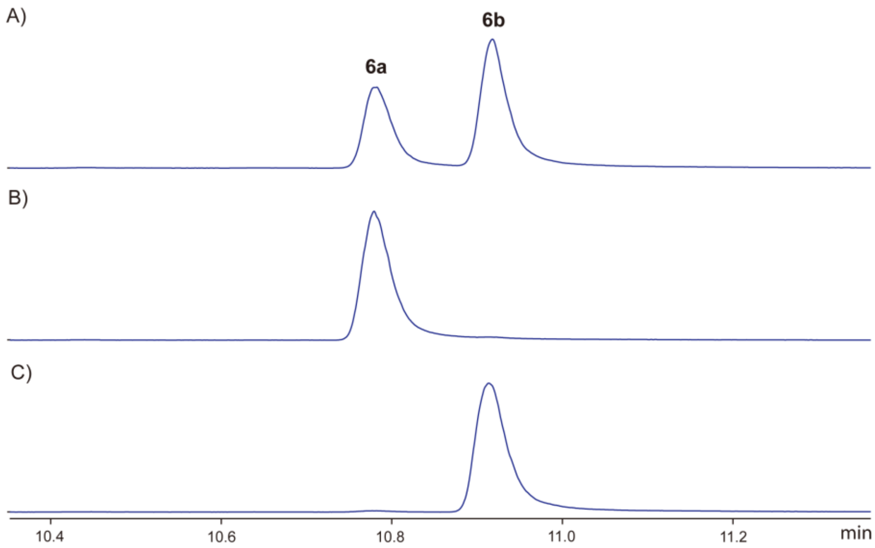

Figure S5: GC analysis of **6a** and **6b** using a chiral stationary phase. A) Pseudoenantiomeric mixture of **6a** and **6b**, B) hexane extract of enantiomerically pure (*R*)-2-Me-LPP after dephosphorylated with CIP, and C) hexane extract of enantiomerically pure (*S*)-2-Me-LPP after dephosphorylated with CIP. Both chromatograms in B) and C) show a high enantiomeric purity (>99% ee), demonstrating that the conversion of the pure enantiomers of **6a** and **6b** into (*R*)- and (*S*)-2-Me-LPP proceeds without loss of enantiomeric purity.

Incubation of enantiomerically pure (*R*)-2-Me-LPP (or (*S*)-2-Me-LPP) in incubation buffer with (or without) 2MIBS

The incubations were performed with 0.3 mg enantiomerically pure (*R*)-2-Me-LPP or (*S*)-2-Me-LPP dissolved in substrate buffer (50 µL; 25 mM NH_4HCO_3) and diluted with incubation buffer (850 µL; 50 mM Tris/HCl, 10 mM $MgCl_2$, 10% glycerol, pH 8.2). 2MIBS protein solution (100 µL) obtained from 100 mL expression culture (or 100 µL elution buffer) was added to the above solutions, followed by incubation with shaking at 30 °C overnight. The crude product was extracted with hexane (500 µL), the extract was dried with $MgSO_4$, and directly analysed by GC/MS (Figure 3 in main text) and GC on a chiral stationary phase (Figures S6–S8).

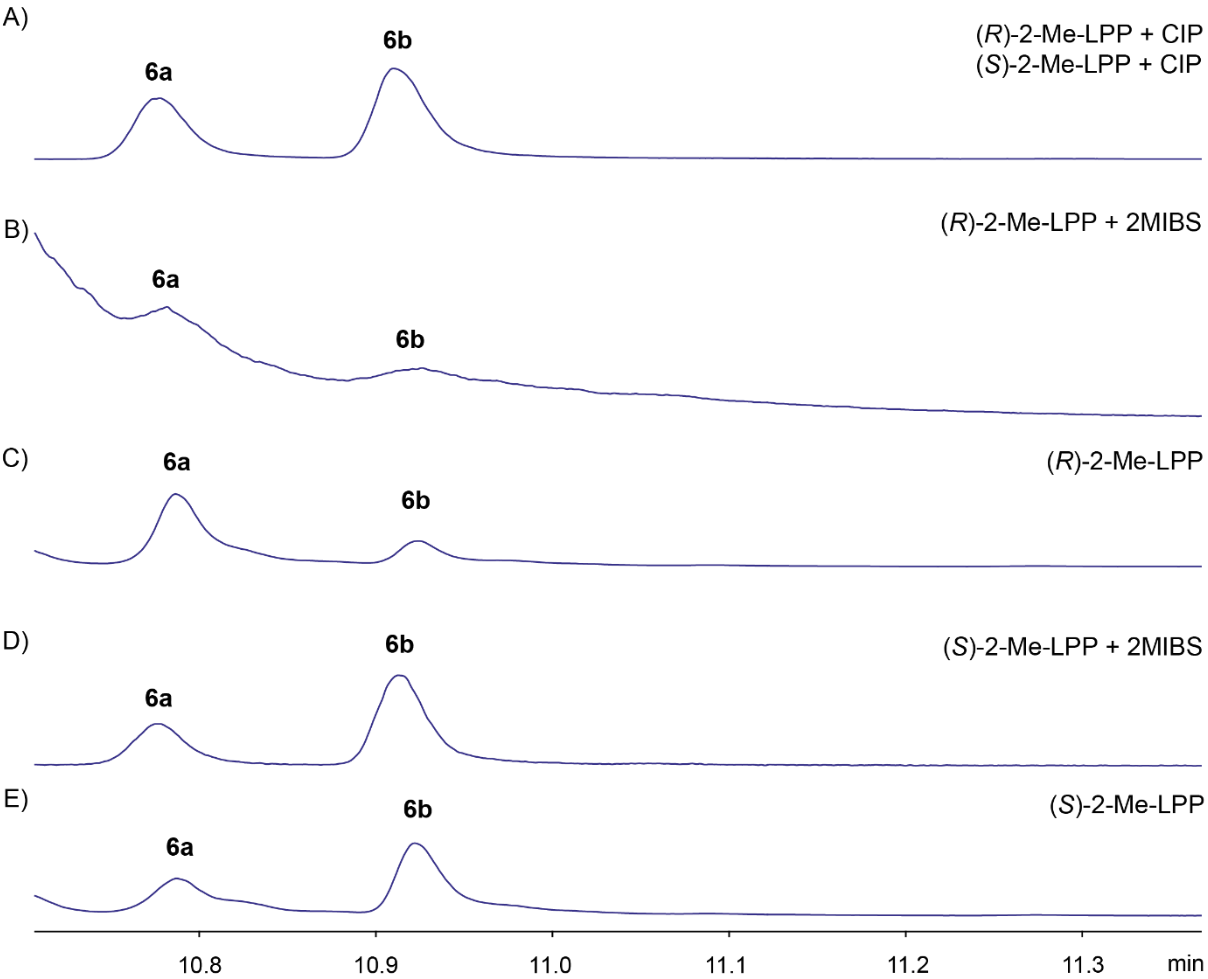

Figure S6: GC analysis of **6a** and **6b** on a chiral stationary phase. Extracts from an incubation of A) a pseudoenantiomeric mixture of (*R*)-2-Me-LPP and (*S*)-2-Me-LPP with CIP, B) enantiomerically pure (*R*)-2-Me-LPP with 2MIBS, C) enantiomerically pure (*R*)-2-Me-LPP without enzyme in incubation buffer, D) enantiomerically pure (*S*)-2-Me-LPP with 2MIBS, and E) enantiomerically pure (*S*)-2-Me-LPP without enzyme in incubation buffer. The nearly unchanged enantiomeric compositions in B) versus C), and in D) versus E), suggest no enzyme participation in the formation of **6** (spontaneous hydrolysis). In part B), the main product 2MIB (**1**) elutes with a large peak directly before the enantiomers of **6** causing a high baseline on the left side of the chromatogram.

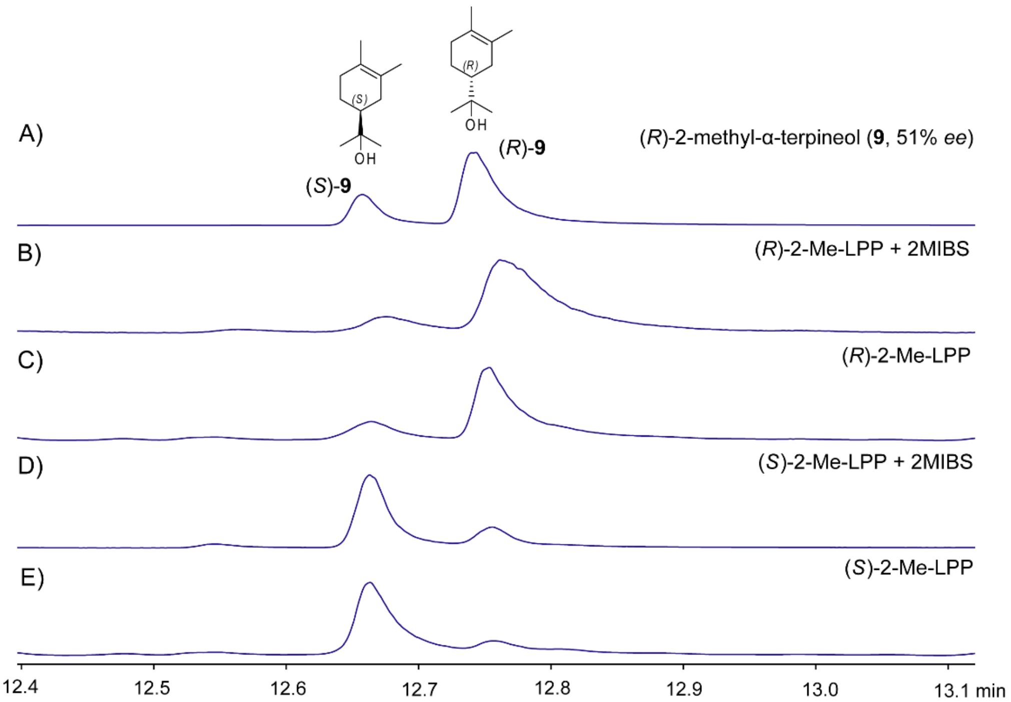

Figure S7: GC analysis of (*R*)-**9** and (*S*)-**9** on a chiral stationary phase. A) Enantiomerically enriched synthetic reference compound (*R*)-**9** (51% ee), extracts from an incubation of B) enantiomerically pure (*R*)-2-Me-LPP with 2MIBS, C) enantiomerically pure (*R*)-2-Me-LPP without enzyme in incubation buffer, D) enantiomerically pure (*S*)-2-Me-LPP with 2MIBS, and E) enantiomerically pure (*S*)-2-Me-LPP without enzyme in incubation buffer. The nearly unchanged enantiomeric compositions in B) versus C), and in D) versus E), suggest no enzyme participation in the formation of **9**.

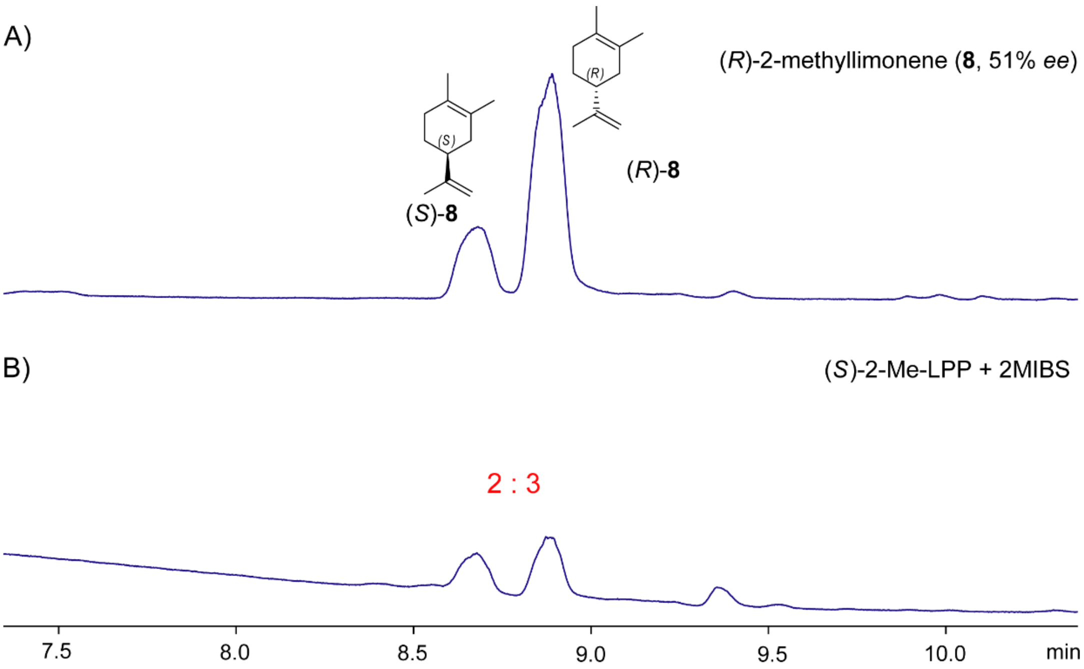

Figure S8: GC analysis of (*R*)-**8** and (*S*)-**8** on a chiral stationary phase. A) Enantiomerically enriched synthetic reference compound (*R*)-**8** (51% ee) and B) extract from an incubation of enantiomerically pure (*S*)-2-Me-LPP with 2MIBS. Compound **8** is not formed without enzyme, and also the ratio of peak integrals of about 2:3 shows participation of the enzyme in the formation of **8** from (*S*)-2-Me-LPP.

Synthesis of enantiomerically enriched (*R*)-2-methyllimonene (8) and (*R*)-2-methyl-α-terpineol (9)

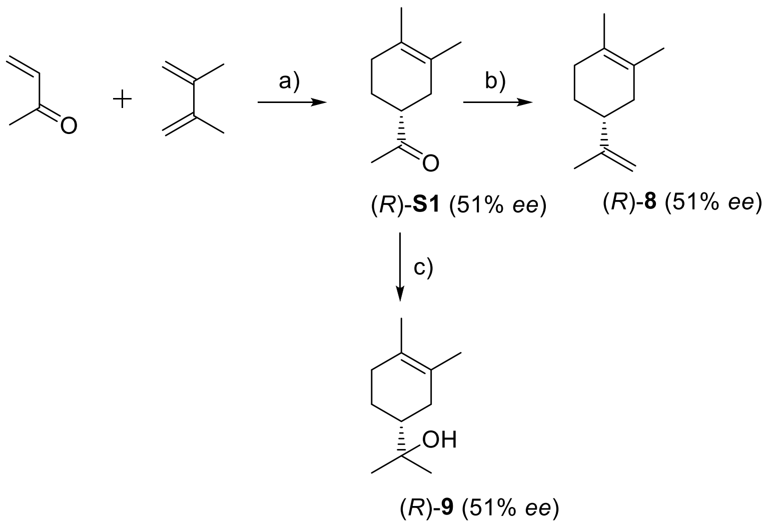

Scheme S1: Synthesis of enantiomerically enriched enzyme products (*R*)-**8** and (*R*)-**9**. Synthetic conditions were: a) (*S*)-(−)-*O*-Tolyl-CBS, triflic acid, CH_2Cl_2, −78 °C to −20 °C, 20 h, 89%; b) CH_3PPh_3I, *n*-BuLi, THF, −78 °C to room temperature, overnight, 64%; c) CH_3MgI, Et_2O, 0 °C to room temperature, 4 h, 47%.

Synthesis of enantiomerically enriched (*R*)-1-(3,4-dimethylcyclohex-3-en-1-yl)ethan-1-one (S1)

The preparation followed a published procedure [9]. (*S*)-(−)-*O*-Tolyl-CBS (1.05 mL, 0.5 M in toluene, 0.53 mmol, 0.27 equiv) was added to a flask, and the solvent toluene was removed under a high vacuum at 50 °C for 2 h. CH_2Cl_2 (6 mL) was then added to the flask. The solution was cooled to −78 °C, followed by the dropwise addition of a freshly prepared triflic acid solution (1.87 mL, 0.2 M in CH_2Cl_2, 0.37 mmol, 0.19 equiv). After stirring at −78 °C for 0.5 h, freshly distilled but-3-en-2-one (140 mg, 2 mmol) was added to the mixture, followed by the slow addition of freshly distilled 2,3-dimethylbuta-1,3-diene (825 mg, 10 mmol, dissolved in 1 mL CH_2Cl_2, 5.00 equiv). The mixture was then stirred at −20 °C for 20 h. Et_3N (0.2 mL, 1.44 mmol, 0.72 equiv) was added to the mixture which was then allowed to warm to room temperature. The solvent was removed under reduced pressure. The enantiomerically enriched target compound (*R*)-**S1** (270 mg, 1.8 mmol, 89%, 51% ee) was obtained as a colourless oil by purification through silica gel chromatography (cyclohexane/ethyl acetate 5:1).

(*R*)-S1: ^{1}H-NMR (700 MHz, $CDCl_3$): δ [ppm] = 2.58-2.53 (m, 1H), 2.16 (s, 3H), 2.15-1.90 (m, 5H), 1.63 (s, 3H), 1.60 (s, 3H), 1.55-1.48 (m, 1H). ^{13}C-NMR (176 MHz, $CDCl_3$): δ [ppm] = 212.01 (C_q), 125.55 (C_q), 124.10 (C_q), 48.46 (CH), 33.26 (CH_2), 31.39 (CH_2), 28.10 (CH_3), 25.49 (CH_2), 19.18 (CH_3), 18.97 (CH_3). TLC [cyclohexane/ethyl acetate (5:1)]: R_f = 0.55. GC (HP5-MS): *I* = 1238. MS (EI, 70 eV) *m*/*z* (%) = 152 (26), 137 (12), 123 (2), 109 (100), 91 (18), 79 (14), 67 (51), 55 (13), 43 (58). $[\alpha]_D^{20}$ = +49.9 (*c* 1.0, $CHCl_3$).

Synthesis of enantiomerically enriched (*R*)-2-methyllimonene (8)

To a THF solution (15 mL, 0 °C) of CH_3PPh_3I (1.43 g, 3.56 mmol, 2.0 equiv) was added *n*-BuLi (2.2 mL, 1.6 M in hexane, 3.55 mmol, 2.0 equiv). The mixture was stirred at 0 °C for 45 min, then cooled to −78 °C. The enantiomerically enriched (*R*)-**S1** (270 mg, 1.77 mmol) was added dropwise to the mixture, followed by stirring overnight without further cooling. The reaction was quenched by the addition of water, and the product was extracted with Et_2O (2 × 20 mL). The solvent was removed under reduced pressure and the enantiomerically enriched product (*R*)-**8** (170 mg, 1.33 mmol, 64%, 51% ee) was purified via flash chromatography (100% pentane) as a colourless oil. GC analysis on a chiral stationary phase was used to determine the enantiomeric excess

(Figure S8A).
(*R*)-8: ^{1}H-NMR (700 MHz, $CDCl_3$): δ [ppm] = 4.72-4.69 (m, 2H), 2.18-2.02 (m, 1H), 1.99-1.87 (m, 3H), 1.79-1.75 (m, 1H), 1.74 (s, 3H), 1.62 (s, 3H), 1.62 (s, 3H), 1.61-1.54 (m, 1H), 1.48-1.37 (m, 1H). ^{13}C-NMR (126 MHz, $CDCl_3$): δ [ppm] = 150.51 (C_q), 125.43 (C_q), 125.25 (C_q), 108.38 (CH_2), 42.14 (CH), 37.34 (CH_2), 32.40 (CH_2), 28.41 (CH_2), 20.94 (CH_3), 19.27 (CH_3), 18.98 (CH_3). TLC [100% pentane]: R_f = 0.66. GC (HP5-MS): *I* = 1132. MS (EI 70 eV) *m*/*z* (%) = 150 (22), 135 (14), 121 (29), 107 (100), 93 (33), 82 (49), 67 (88), 53 (11), 41 (24). $[\alpha]_D^{20}$ = +2.6 (*c* 1.0, $CHCl_3$).

Synthesis of enantiomerically enriched (*R*)-2-methyl-α-terpineol (9)

Magnesium turnings (108 mg, 4.50 mmol, 3.6 equiv) and Et_2O (1.5 mL) were added to a flame dried flask, followed by the addition of a small portion of CH_3I. The reaction was triggered by gentle warming with a heat gun, and then CH_3I (532 mg, 3.75 mmol, dissolved in 0.5 mL Et_2O, 3.0 equiv) was added dropwise to keep the solution at reflux. After completion of the addition of CH_3I, the mixture was stirred at room temperature for another 0.5 h. To an Et_2O (4 mL, 0 °C) solution of the enantiomerically enriched (*R*)-**S1** (190 mg, 1.25 mmol) was added the freshly prepared CH_3MgI solution dropwise. The mixture was stirred at room temperature for 4 h. The reaction was quenched with saturated aq. NH_4Cl. The product was extracted with Et_2O (3 × 30 mL), and the solvent was removed under reduced pressure. The enantiomerically enriched product (*R*)-**9** (100 mg, 0.59 mmol, 47%, 51% ee) was purified via silica gel chromatography (pentane/Et_2O 1:1) and obtained as a colourless oil. GC analysis using a chiral stationary phase was used to determine the enantiomeric excess (Figure S7A).
(*R*)-9: ^{1}H-NMR (500 MHz, $CDCl_3$): δ [ppm] = 2.11-1.99 (m, 1H), 2.01-1.89 (m, 2H), 1.87-1.76 (m, 2H), 1.65-1.58 (m, 6H), 1.57-1.50 (m, 1H), 1.40 (s, 1H), 1.25-1.20 (m, 1H), 1.20 (s, 3H), 1.18 (s, 3H). ^{13}C -NMR (126 MHz, $CDCl_3$): δ [ppm] = 125.73 (C_q), 125.12 (C_q), 72.86 (C_q), 45.98 (CH), 33.38 (CH_3), 32.83 (CH_3), 27.48 (CH_2), 26.51 (CH_2), 24.47 (CH_2), 19.40 (CH_3), 18.93 (CH_3) [10]. TLC [pentane/Et_2O = (1:1)]: R_f = 0.50. GC (HP5-MS): *I* = 1294. . MS (EI 70 eV) *m*/*z* (%) = 168 (2), 150 (27), 135 (36), 121 (6), 107 (100), 93 (21), 81 (14), 67 (24), 59 (59), 43 (35). $[\alpha]_D^{20}$ = +74 (*c* 1.0, $CHCl_3$).

References

1. Fulmer, G. R.; Miller, A. J. M.; Sherden, N. H.; Gottlieb, H. E.; Nudelman, A.; Stoltz, B. M.; Bercaw, J. E.; Goldberg, K. I. *Organometallics,* **2010,** *29,* 2176–2179.
2. Dickschat, J. S.; Nawrath, T.; Thiel, V.; Kunze, B.; Müller, R.; Schulz, S. *Angew. Chem. Int. Ed.,* **2007,** *46,* 8287–8290.
3. Le, T. C.; Chauhan, K. R. *Nat. Prod. Commun.,* **2014,** *9,* 297–298.
4. Hoye, T. R.; Jeffrey, C. S.; Shao, F. *Nat. Protoc.,* **2007,** *2,* 2451–2458.
5. Thulasiram, H. V.; Phan, R. M.; Rivera, S. B.; Poulter, C. D. *J. Org. Chem.,* **2006,** *71,* 1739–1741.
6. Keller, R. K.; Thompson, R. *J. Chromatogr. A,* **1993,** *645,* 161–167.
7. Gu, B.; Dickschat, J. S. *Chem. Commun.,* **2022,** *58,* 4316–4319.
8. Brock, N. L.; Ravella, S. R.; Schulz, S.; Dickschat, J. S. *Angew. Chem. Int. Ed.,* **2013,** *52,* 2100–2104.
9. Ryu, D. H.; Lee, T. W.; Corey, E. J. *J. Am. Chem. Soc.,* **2002,** *124,* 9992–9993.
10. Chou, W. K.; Gould, C. A.; Cane, D. E. *J. Antibiot.,* **2017,** *70,* 625-631.
